# Observation of enhanced nanoscale creep flow of crystalline metals enabled by controlling surface wettability

**DOI:** 10.1038/s41467-022-35703-6

**Published:** 2022-12-26

**Authors:** Jun-Xiang Xiang, Ze Liu

**Affiliations:** 1 Department of Engineering Mechanics, School of Civil Engineering, Wuhan University, 430072 Wuhan, Hubei China; 2 State Key Laboratory of Water Resources & Hydropower Engineering Science, Wuhan University, 430072 Wuhan, Hubei China; 3 The Institute of Technological Science, Wuhan University, 430072 Wuhan, Hubei China

**Keywords:** Synthesis and processing, Surfaces, interfaces and thin films, Surface patterning

## Abstract

Understanding and controlling interface friction are central to many science and engineering applications. However, frictional sliding is closely related to adhesion, surface roughness, surface chemistry, mechanical deformation of contact solids, which poses the major challenge to experimental studying and theoretical modeling of friction. Here, by exploiting the recent developed thermomechanical nanomolding technique, we present a simple strategy to decouple the interplay between surface chemistry, plastic deformation, and interface friction by monitoring the nanoscale creep flow of metals in nanochannels. We show that superhydrophobic nanochannels outperforming hydrophilic nanochannels can be up to orders of magnitude in terms of creep flow rate. The comparative experimental study on pressure and temperature dependent nanomolding efficiency uncovers that the enhanced creep flow rate originates from diffusion-based deformation mechanism as well as the superhydrophobic surface induced boundary slip. Moreover, our results reveal that there exists a temperature-dependent critical pressure below which the traditional lubrication methods to reduce friction will break down. Our findings not only provide insights into the understanding of mechanical deformation and nanotribology, but also show a general and practical technique for studying the fundamental processes of frictional motion. Finally, we anticipate that the increased molding efficiency could facilitate the application of nanoimprinting/nanomolding.

## Abstract (web summary)

Solid-solid interface friction usually becomes the bottleneck at micro/nanoscale. Here, Xiang and Liu show that the nanoscale creep flow rate of crystalline metals can increase by orders of magnitude when the contact metal is in diffusion deformation and the thermally activated boundary slip is active.

## Introduction

Understanding and controlling interface friction play an important role in many science and engineering applications^1–4^, typically such as micro- and nanomachines^2,5^, biological molecular motors^5,6^, magnetic storage and recording systems^7,8^, fluids transport^9–11^, and manufacturing^12–14^. Especially, when the characteristic size of a system shrinks to the micro and nanoscale, the drastically increased specific surface/interface area usually makes friction a bottleneck problem^12,15,16^. For example, it was observed that the friction in metal forming increases with decreasing specimen size^12^, and a microelectromechanical systems (MEMS) actuator operated in vacuum fails very quickly^16^. Considering that in general the strong interfacial adhesion induces high friction, lubricant-coated or hydrophobic surfaces are introduced to reduce the friction resistance^8,17^. In the past two decades, evidence of boundary slip between liquid and solid at the nanoscale^18–25^ has been demonstrated by using superhydrophobic^26–31^ or lubricant-coated surfaces^17,32–34^. However, the solid-solid interface friction is much more complex, which significantly depends on adhesion^1,2^, surface chemistry^7,35^, surface structure or roughness^15^, and complex mechanical deformation of solids^2,4,36,37^. At present, understanding and predicting the frictional response of solid-solid contact interface in countermotion by decoupling the effects of surface chemistry, surface roughness and the deformation of solids remain a challenge because of the lack of effective tribological and lubrication techniques^2,4,36^. Recently, an advanced thermomechanical nanomolding technique^13,38^ has permitted us to decouple the dislocation and diffusion-based deformation mechanisms of crystalline metals, and correlate them with the specific microscale physical processes^39–41^.

In this article, by thermally compressing crystalline metals onto anodic aluminum oxide (AAO) nanomolds with smooth nanochannels, the interplay between surface chemistry, plastic deformation, and interface friction is decoupled and quantified by directly comparing the length difference of metals flowing into nanochannels with pristine and silanization coated surfaces. The designed comparative experiments allow us to observe that superhydrophobic nanochannels outperforming hydrophilic nanochannels can be up to orders of magnitude in terms of creep flow rate, depending on the molding temperature and pressure, and to obtain a fundamental understanding of the pressure and temperature dependence of friction, which originates from diffusion-based deformation mechanism as well as the superhydrophobic surface induced boundary slip.

## Results

### Quantifying the effect of surface wettability on interface friction by thermomechanical nanomolding

**Fig. 1:**
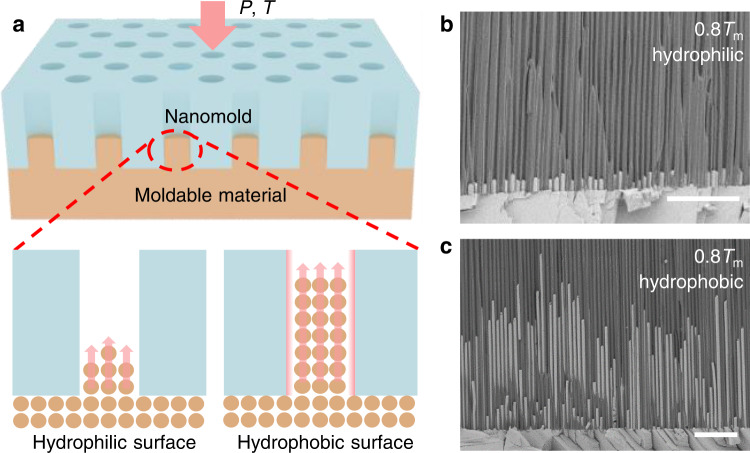
Quantifying interface friction by thermomechanical nanomolding (TMNM). **a** Sketch of typical experimental set-up for studying the effect of surface wettability on interface friction. **b**–**c** SEM image of Bi nanowires prepared by nanomolding at 0.8*T*_m_ and with hydrophilic (**b**) and hydrophobic (**c**) surfaces, respectively. the loading rate and the maximum molding pressure were 10 MPa/s and 250 MPa, respectively. Scale bars: 5 μm.

To quantify the effect of surface chemistry on interface friction of contact solids in countermotion at the nanoscale, thermomechanical nanomolding technique is applied^38–40^ (Fig. 1), where a crystalline metal is compressed against a nanomold at a controlled temperature. The crystalline metal will creep flow into the nanochannels of the nanomold once the loading force is higher than the flow resistance force. Subsequently, by simply measuring the length difference of the metal filled in hydrophilic and hydrophobic nanochannels, the effect of surface wettability can be quantified by

1 $$\lambda=\frac{{L}_{2}-{L}_{0}}{{L}_{1}-{L}_{0}}$$

where *L*_0_ is the initial filled length before the comparative experiments in Fig. 1, ${L}_{2}$ and ${L}_{1}$ are the growth length under the same molding conditions but with hydrophobic and hydrophilic nanochannels, respectively. The AAO nanomolds are intrinsic hydrophilic (Supplementary Fig. 1a). The superhydrophobic AAO surface was realized by silanization treatment (see Methods) and characterized by the contact angle measurement (Supplementary Fig. 1b). The nanomold walls are very smooth (with RMS of ~0.370 nm, Supplementary Fig. 2a–c) and they show negligible changes after silanization coating (with RMS of ~0.356 nm, Supplementary Fig. 2d–f), therefore, we can safely exclude the influence of roughness in our study^42^. Since the AAO nanomold is made of hard ceramic material (there is no observable dimension changes in the filled metal nanorods, Fig. 1b, c) and all other conditions are kept the same in the comparative experiments, the dimensionless parameter $\lambda$ is only determined by the deformation mechanism of the metal and the interface friction.

**Fig. 2:**
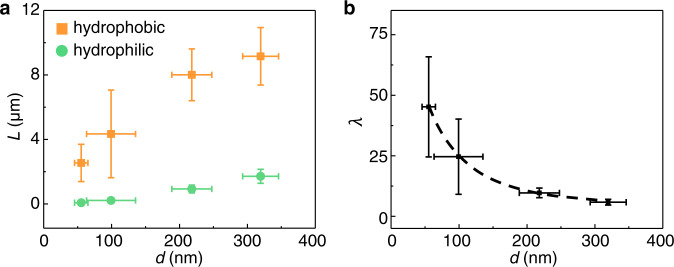
Nanochannel size dependent creep flow of Bi. **a** Measured length of Bi nanowires versus cavity size, where the Bi nanowires were molded by using AAO nanomolds with cavity size of ~55, 100, 220 and 320 nm, respectively. The loading pressure was increased from zero to 300 MPa with a loading rate of 3 MPa/s. The molding temperature was set as 0.68*T*_m_. **b** Size dependent surface chemistry effect by calculating λ based on the data in (**a**) and by using Eq. (1). The error bars are the standard deviation calculated from the length of at least 10 nanorods. Source data are provided as a Source Data file.

On the basis of the procedures in Fig. 1a, we found that the length of Bi nanowires grown from superhydrophobic AAO nanochannels (after silanization treatment) can be much longer than that grown from pristine AAO nanochannels (Fig. 1b, c). Considering that the plastic deformation of metals is usually diffusion based at temperature larger than ~0.5*T*_m_^40^, and the molding temperature in Fig. 1b, c is 0.8*T*_m_, we attribute the enhanced diffusion creep rate to the decrease of interface friction caused by superhydrophobic nanochannel. A similar mechanism has been demonstrated in the flow of liquids such as polymer melts at superhydrophobic^26–31^ or lubricant-coated nanochannels^17,32–34^, but has never been achieved in crystals. Further evidence is from the size-dependent growth length of metal nanorods. Typical results are shown in Fig. 2, where thermomechanical nanomolding of Bi was carried out at 0.68*T*_m_ by using AAO nanomolds with different cavity size. By comparison with pristine AAO nanochannels, the silanization-treated AAO nanochannels show enhanced diffusion creep flow rate for all the used cavity sizes (Fig. 2a). Figure 2b shows that the promotion effect by using superhydrophobic nanochannels decreases as the cavity size (*d*) increases. Considering that the same molding temperature and pressure ensure the same deformation mechanism in the metal, and the specific surface area (i.e., the ratio of surface to volume) is inversely proportional to the nanochannel size, it is reasonable to conclude that it is the reduced interface friction caused by silanization treatment that leads to the smaller the size, the more enhanced creep flow rate.

### Interplay between the surface chemistry, plastic deformation, and interface friction

**Fig. 3:**
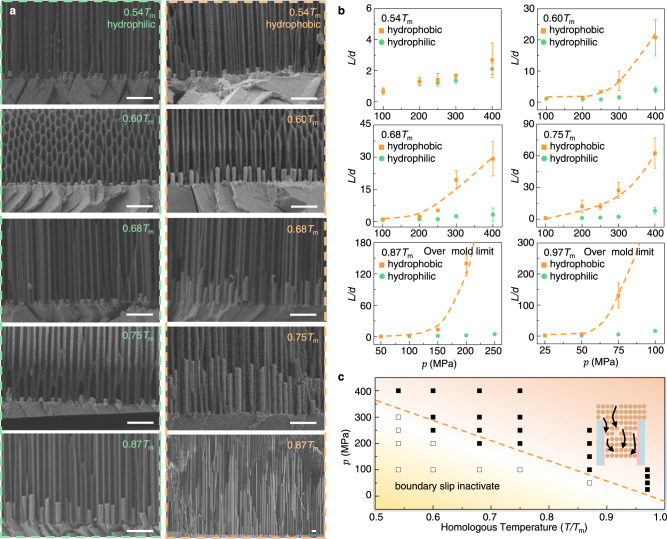
Temperature dependence of higher creep flow of Bi enabled by superhydrophobic surface. **a** SEM characterization of Bi nanowires molded with pristine (left figures) and silanization-treated AAO nanomolds (right figures, see “Methods”"), where the molding pressure was linearly increased from zero to 250 MPa with a loading rate of 10 MPa/s, and AAO nanomolds with cavity size of *d* = 300 nm were used. Scale bars: 2 μm. **b** Measured length of Bi nanowires molded at different pressure and temperature. The dashed lines are given as guides to the eye. **c** The molding pressure versus the molding temperature. The solid and open squares correspond to whether the length difference of Bi nanowires molded with pristine and silanization-treated AAO nanomolds is significantly distinguishable (two times difference) or indistinguishable, respectively. The dashed line is fitted based on Eq. (3). The error bars are the standard deviation calculated from the length of at least 10 nanorods. Source data are provided as a Source Data file.

To further reveal the interplay between surface chemistry, plastic deformation and interface friction, thermomechanical nanomolding of Bi was carried out at various temperature and pressure by following the strategy in Fig. 1. The molded samples by using pristine AAO nanomolds are set as references (left figures of Fig. 3a). It is observed that the length of Bi nanowires molded with superhydrophobic nanomolds are much longer if the molding temperature is high, e.g. *T* > 0.5*T*_m_ (right figures of Fig. 3a). However, when the molding temperature is low such as 0.4*T*_m_, even if the molding pressure is increased to 600 MPa, there is a negligible length difference in the molded Bi nanowires whether the AAO nanomolds is treated with silanization or not (Supplementary Fig. 3). These observations suggest that the influence of surface chemistry on interface friction at the nanoscale is closely related with the deformation mechanism of contact solids in countermotion. During steady-state thermomechanical nanomolding, the growth of metal nanorods originates from the plastic deformation of bulk metals, where the input work by the external load is balanced by the plastic deformation work plus the interface friction-induced energy dissipation. When the molding temperature is low, typically lower than 0.5*T*_m_, the deformation mechanism of crystalline metals is based on dislocation movement^38,40,43^. The plastic deformation work caused by dislocation movement is essentially related to internal friction, which corresponds to the relative motion between crystals on both sides of a sliding crystal plane (i.e., slip plane). The internal friction could be much larger than the metal–mold interface friction because the former involves in breaking and reformation of metallic bonds. Therefore, the energy dissipation induced by interface friction may be negligible by comparison with that caused by internal friction through dislocation movement, then the superhydrophobic surface enhanced high creep rate would disappear at the dislocation motion dominated temperature range, this is exactly what we observed in experiments (Fig. 3 and Supplementary Fig. 3).

On the other hand, when the molding temperature is increased to larger than ~0.5*T*_m_, the promotion of superhydrophobic surface on the growth of Bi nanowires rapidly increases as temperature (Fig. 3a, b). Considering that the diffusion-assisted dislocation motion (e.g. dislocation creep) and diffusion flow (e.g. the Nabarro-Herring creep^44^ and Coble creep^45^) take over the deformation mechanism of metals at temperatures larger than ~0.5*T*_m_^13,45,46^, we attribute the promotion effect to the interplay between surface chemistry and diffusion-based deformation of solids. Because the diffusion-based creep flow of metals is similar to viscous fluid^44^, the enhanced nanoscale creep flow in crystalline metals caused by superhydrophobic surface might be similar to the superhydrophobic surface-induced boundary slip in fluid-solid systems^47–49^. The viscous flow in channels usually exhibits a parabolic velocity distribution, that is, the velocity at the metal–mold interface is the smallest (it is zero for “no-slip” boundary). Then the superhydryphobic surface-induced boundary slip (or friction reduction) will increase the creep flow rate, as a result, the metal nanowires grown from suerphydrophobic channels are longer than those grown from hydrophilic channels (Fig. 3a). Similar boundary slip mechanism has been verified in hydrophobic surface enhanced flow of liquids^17,26–31^.

It should be noted that adhesion hysteresis is usually also related to surface chemistry^50^, and it is difficult to completely eliminate its influence on interface friction. However, considering that adhesion hysteresis is rate sensitive^51,52^, we can investigate its contribution to interface friction by drastically varying the loading and/or unloading rate. Typical results are shown in Supplementary Fig. 4, it is observed that the length of Bi nanorods increases as the loading/unloading rate decreases by three orders of magnitude, regardless of whether the nanomold walls are silanization coated or not (Supplementary Fig. 4b). This is reasonable since the molding temperature and maximum molding pressure were kept constant in the experiments (Supplementary Fig. 4a), a lower loading/unloading rate will result in a longer growth time of Bi nanorods under pressure. However, by calculating the length difference of Bi nanorods molded with pristine and silanization coated nanomolds by using Eq. (1), we observed that although the loading/unloading rate has changed by three orders of magnitude, there is no notable change in $\lambda$ value (Supplementary Fig. 4c). Therefore, we conclude that though adhesion hysteresis is generally correlated with interface friction, it is not the dominant mechanism in our observations (the considerably increased creep rate).

### Thermally activated boundary slip

Considering that the frictional response of tribological contact generally involves in detachment and reattachment of multiple microscopic bonds between the surfaces in relative motion, and thermal excitations can provide sufficient energy to overcome local barriers and enable boundary slip or friction reduction^1–4,36^, in the temperature range based on diffusion deformation mechanism, superhydrophobic surface induced boundary slip or the transition rate (*w*) between detachment and reattachment should rely on the energy barrier (*E*) as *w*∝exp(−*E*/*k*_B_*T*)^21,36,53^, where *T* is the absolute temperature, *k*_B_ is the Boltzmann’s constant. Because the energy barrier can be biased towards the pressure gradient, we can finally obtain the transition rate between the states (before and after slip) as^36^

2 $$w={f}_{0}\exp \left(-\frac{E-pV}{{k}_{B}T}\right)$$

where *f*_0_ is the attempt frequency setting the characteristic timescale of thermal relaxation processes^53^. *V* is the activation volume and *p* is the pressure exerted on the activation volume, which directly depends on the molding pressure^54,55^. Experimentally, the critical molding pressure (*p*_c_) corresponds to the case that it is just able to distinguish the length difference of metal nanorods molded with and without silanization treated AAO nanomolds. If denoting the corresponding transition rate as *w*_0_, then based on Eq. (2), we have

3 $${p}_{c}=\frac{E}{V}-\alpha T$$

where *α* = −*k*_B_/*V* ln(*w*_0_/*f*_0_) > 0. Equation (3) predicts that the critical molding pressure linearly decreases as the temperature increasing. To test the theoretical prediction, we displayed the molding efficiency (quantified by the aspect ratio of molded metal nanowires) in the parameter space of temperature and pressure on the basis of the comparative experimental results in Fig. 3a, b (Fig. 3c), where the solid and open squares represent whether the length difference of Bi nanowires molded with and without silanization treated AAO nanomolds is significantly distinguishable or not. It is observed that there exists a critical pressure (*p*_cr_) below which the difference of the aspect ratio of Bi nanowires molded with pristine and silanization-treated AAO nanomolds is indistinguishable, which suggests the breakdown of interface lubrication. When the molding pressure is higher than *p*_cr_, the aspect ratio of Bi nanowires molded with silanization-treated AAO nanomolds shots up over one order of magnitude compared with that molded with pristine AAO nanomolds (Fig. 3b). If taking the solid squares just above the open squares as the critical pressure, it is clear that the critical pressure can be well fitted with Eq. (3) (dotted line in Fig. 3c).

### Universality of superhydrophobic surface enhanced creep flow of crystalline metals

**Fig. 4:**
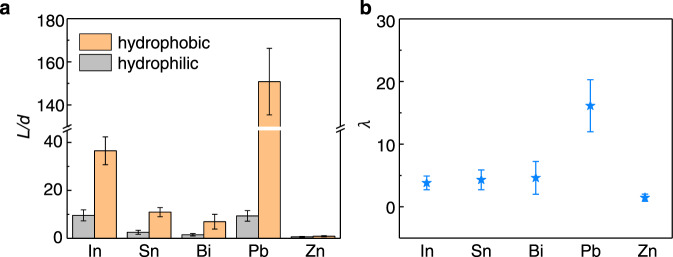
Generality of the enhanced molding efficiency in TMNM of crystalline metals by using superhydrophobic nanomolds. **a** Examples of superhydrophobic surface-assisted nanomolding of metals with different crystalline structures at 0.6*T*_m_, which is enabled by TMNM with silanization treated AAO nanomolds, i.e. In (Monoclinic), Sn (Tetragonal), Bi (Triclinic), Pb (Cubic), and Zn (Hexagonal). The loading pressure was increased from zero to 300 MPa with a constant loading rate of 10 MPa/s. **b** Calculating the value of λ based on (**a**) and by using Eq. (1). The error bars are the standard deviation calculated from the length of nanorods in Supplementary Fig. 6. Source data are provided as a Source Data file.

The above experiments clearly show that superhydrophobic surface-enhanced creep flow of Bi originates from diffusion-based deformation mechanism as well as superhydrophobic surface-induced boundary slip. Considering that the plastic deformation ability of metals significantly depends on the crystalline structure, and considering the thermal stability of silanization coating (Supplementary Fig. 5a), we selected five low melting point metals with different crystalline structures for comparative experiments, i.e., In (Monoclinic), Sn (Tetragonal), Bi (Triclinic), Pb (Cubic), and Zn (Hexagonal). Typical results are shown in Fig. 4a and Supplementary Fig. 6, where the molding temperature and molding pressure for all the metals were kept the same, i.e., 0.6*T*_m_ and 300 MPa. It is observed that Pb shows the highest enhancement of creep flow (Fig. 4b). This is reasonable since the cubic crystalline structure possesses the most slip systems and the deformation ability is the highest^43^. It is also noted that Zn shows negligible enhancement at 0.6*T*_m_ (Fig. 4b), but significant enhancement at 0.68*T*_m_ and 0.75*T*_m_ (with the same molding pressure of 300 MPa, Supplementary Fig. 7), which we attribute to the crystalline structure affected deformation transition temperature. More evidence is shown in Supplementary Fig. 7–9, where the temperature-dependent critical pressure for enhanced creep flow of Zn and Pb enabled by the superhydrophobic surface is identified. As anticipated, for a given temperature (larger than the deformation transition temperature), the critical pressure required for the creep flow enhancement of Zn is much higher (Supplementary Fig. 9a).

It is noted that when thermomechanical nanomolding of metals with high melting temperature (e.g., Au, Ag, Cu), the molding efficiency by using superhydrophobic nanomolds is still improved but much weaker, especially for Ag and Cu (Supplementary Fig. 10). Considering that the boiling point of used silanization coating is 250 °C, and the diffusion deformation-based molding temperature required for Au, Ag and Cu is much higher, for example, 0.6*T*_m_ of Ag is ~468 °C, we attribute the reduction of superhydrophobic surface enhanced creep flow in Au, Ag and Cu to the partial decomposition of silanization coating. XPS analysis of silanization-coated AAO nanomolds which were pre-heated at different temperatures clearly show that when the temperature is low, such as 293 and 473 K, the characteristic peaks of silanization were observed (Supplementary Fig. 5), that is, C-Si @ 283.3 eV, C–C @ 284.0 eV, C–O @ 285.1 eV, CF_2_–CH_2_ @ 290.2 eV, CF_2_ @ 290.8 eV, and CF_3_ @ 292.8 eV^56^. However, when the temperature is increased to 673 K or above, the characteristic peaks of CF_2_–CH_2_ (290.2 eV), CF_2_ (290.8 eV), and CF_3_ (292.8 eV) disappear, at the meantime, the peak of C–O (285.1 eV) is significantly strengthened (refer to Supplementary Table S1 for the sample surface relative atomic concentration), which indicates that the silanization coating will decompose at high temperature, and the fluorine atom disappears while the oxygen atom content increases. Considering that Ag and Cu are more reactive with oxygen, the observed negligible effect of hydrophobicity on the creep flow of Ag and Cu (by comparison with Au) is attributed to the stronger chemical affinity between Ag (Cu) and partially decomposed silanization coating.

## Discussion

Microscopically, the deformation mechanisms are closely correlated with microstructures (e.g., lattice defects, grain boundaries, etc.). For example, the low-temperature deformation mechanism of metals is usually based on dislocation movement, where the line defects (i.e., dislocations) are the carrier of plastic deformation. While the high-temperature deformation mechanism is usually based on diffusion of defects, such as vacancy diffusion and dislocation climb. It should be pointed out that the normalized deformation mechanism transition temperature in textbook (~0.5*T*_m_) is only a general guideline. Our experiments on nanomolding of Zn indicate a much higher transition temperature (Supplementary Fig. 9a), whether this observation is common for closely packed hexagonal crystals needs further study. On the other hand, although the conclusion obtained from the comparative experiments should be insensitive to specific microscale physical processes behind the deformation mechanisms because we only change one processing condition and all other conditions are kept the same in each comparative experiment, the actual governing mechanism of crystalline metals at different temperatures and stresses is worth for future research. For example, for metals with low stacking fault energy such as silver, twinning deformation is also an important deformation mechanism. However, twins are rarely observed in our experiments; We observed high density of dislocations in Ag nanorods molded at 0.4*T*_m_. When increasing the temperature to 0.6*T*_m_, perfect crystal structure (without notable dislocations) is observed (Supplementary Fig. 11). Tin is a typical metal with potential strain-induced phase transformation, even when we molded a bulk Sn with forming pressure of 300 MPa, no change in crystalline structure was observed before and after nanomolding (Supplementary Fig. 12). Therefore, under what conditions twinning or phase transition will occur may be a promising line of further study.

Besides, the feedstock is usually polycrystalline, the loading direction with respect to crystallographic orientation will affect the creep flow^40^, this is why the molded metal nanorods in one sample do not have the same length (e.g., Supplementary Fig. 13). However, when we characterized molded samples in a range much larger than the grain size, the silanization coated nanomold results in longer nanorods in the whole range. Typical results for nanomolding of Sn are shown in Supplementary Fig. 13. In fact, our previous study and others show that even though the feedstock is polycrystalline, the molded metal nanorods prefer to grow along a crystalline orientation with lower surface energy, for example, the molded FCC metal nanorods are almost all along [110] direction, independent on the substrate crystalline orientation^46,57^. More evidence is from the XRD spectra of Sn before and after nanomolding (Supplementary Fig. 12), which shows that the signal of crystalline face (200) with the lowest surface energy^58^ is significantly enhanced after nanomolding.

Remarkably, a simple method is proposed to experimentally quantify the interplay between surface chemistry, the deformation of contact solids in countermotion, and the interface friction by monitoring the growth length of metal nanowires at controlled temperature and pressure, which enables us to discover that the effects of surface wettability and the plastic deformation of contact metal on interface friction are strongly coupled, and there exists a temperature-dependent critical pressure below which the traditional lubrication methods will break down. We further demonstrate that in the diffusion deformation-based temperature range, a superhydrophobic surface can considerably reduce the creep flow resistance of crystalline metals in nanochannels. Finally, we show that the superhydrophobic surface enhanced creep flow in crystalline metals possesses an evident universality, but the enhancement of creep rate is closely related to the crystalline structure. We anticipate that these findings can not only deepen the understanding of the fundamental mechanisms of mechanical deformation and tribology at nanoscale, but also provide a potential solution to the miniaturization challenge in the metal forming society^12^, especially guide the molding-based nanofabrication and promote the wide application of metal nanostructures^13^.

## Methods

### Materials

The Anodic Aluminum Oxide (AAO) template was purchased from Shenzhen Topmembranes Technology Co. Ltd. Bulk metals (with purity of 99.99%) were purchased from Beijing Hezong Science & Technology Co., Ltd. 1H, 1H, 2H, 2H perfluorodecyltriethoxysilane (silanization) was purchased from Sinopharm Chemical Reagent Co., Ltd.

### TMNM of crystalline metals

All the nanomolding experiments were carried out in a universal testing machine equipped with a home-built heating unit (CMT6105). Taking TMNM of Bi for example (Fig. 1b, c, Figs. 2, 3), Bi rods with similar weight (~0.07 g) were first cut from the same rod, and then pre-compressed at 200 °C to obtain thin flat discs (with diameter and thickness of ~6 mm and ~0.25 mm, respectively). The load was linearly increased from zero to 1.5 kN at a loading rate of 100 N/s. After grinding and cleaning, the Bi discs were compressed onto AAO nanomolds at a loading rate of 100 N/s to 1.5 kN to obtain an initial filling length (*L*_0_ ~ 170 nm, Supplementary Fig. 14). The formed Bi/AAO nanomolds combinations were following chemically modified with silanization by molecular vapor depositing at 100 °C for 2 h. Finally, compression forming of Bi/AAO nanomolds combinations with and without silanization treatment was carried out at the same conditions by following the procedures in Fig. 1. The lengths of nanowires in the center of each sample were characterized under SEM (Fig. 1b, c, Figs. 2, 3).

### Characterization

The length of nanowires was measured by using a scanning electron microscopy (SEM, Zeiss Sigma 500). Characterization of the surface wettability of AAO nanomold before and after silanization treatment was carried out by measuring the contact angle under an optical microscope (VHX-5000, KEYENCE). X-ray photoelectron spectroscopy (XPS, Thermo Fisher ESCALAB250Xi) was carried out on a Thermo Fisher ESCALAB250Xi. The roughness of AAO channel was characterized by an atomic force microscopy (AFM, Bruker Dimension Icon.) The crystalline structure was confirmed by a X-ray diffractometer (XRD, TDM-10, Tongda).

## Supplementary information

Supplementary Information

## Data Availability

The data that support the findings of this study are available from the corresponding author upon request. Source data are provided with this paper.

## Source data

Source Data
