# Supplementary material for: Observation of enhanced nanoscale creep flow of crystalline metals enabled by controlling surface wettability
Source: Nat Commun. 2022 Dec 26;13:7943. doi: 10.1038/s41467-022-35703-6 (PMC9792587; doi:10.1038/s41467-022-35703-6)
Supplement: Supplementary file 1 — Supplementary Information [file 41467_2022_35703_MOESM1_ESM.pdf]

Supplemental Material for

Observation of enhanced nanoscale creep flow of crystalline metals enabled by controlling surface wettability

Jun-Xiang Xiang[1], Ze Liu[1,2,3*]

[1] Department of Engineering Mechanics, School of Civil Engineering, Wuhan University, Wuhan, Hubei 430072, China

[2] State Key Laboratory of Water Resources & Hydropower Engineering Science, Wuhan University, Wuhan, Hubei 430072, China.

[3] The Institute of Technological Science, Wuhan University, Wuhan, Hubei 430072, China

*Corresponding author. Email: ze.liu@whu.edu.cn

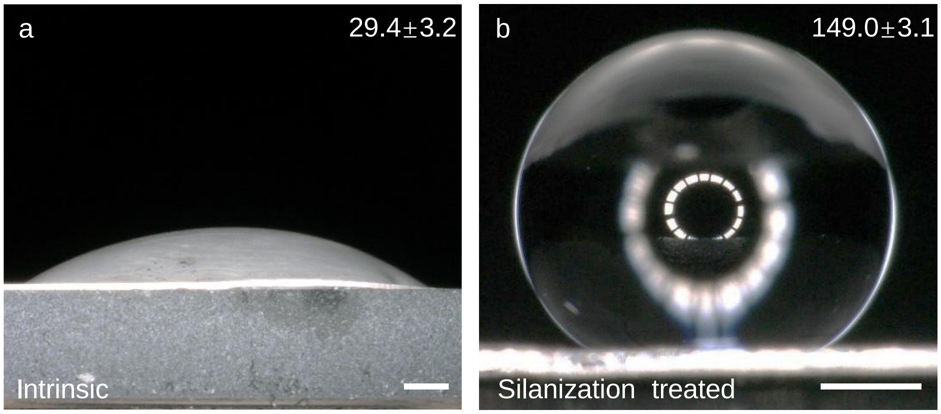

Supplementary Fig. 1 | Wetting of 3 μL water droplets on AAO molds. (**a**) Intrinsic contact angle of AAO mold. (**b**) Contact angle of AAO mold after silanization treatment. Scale bars: 0.5 mm.

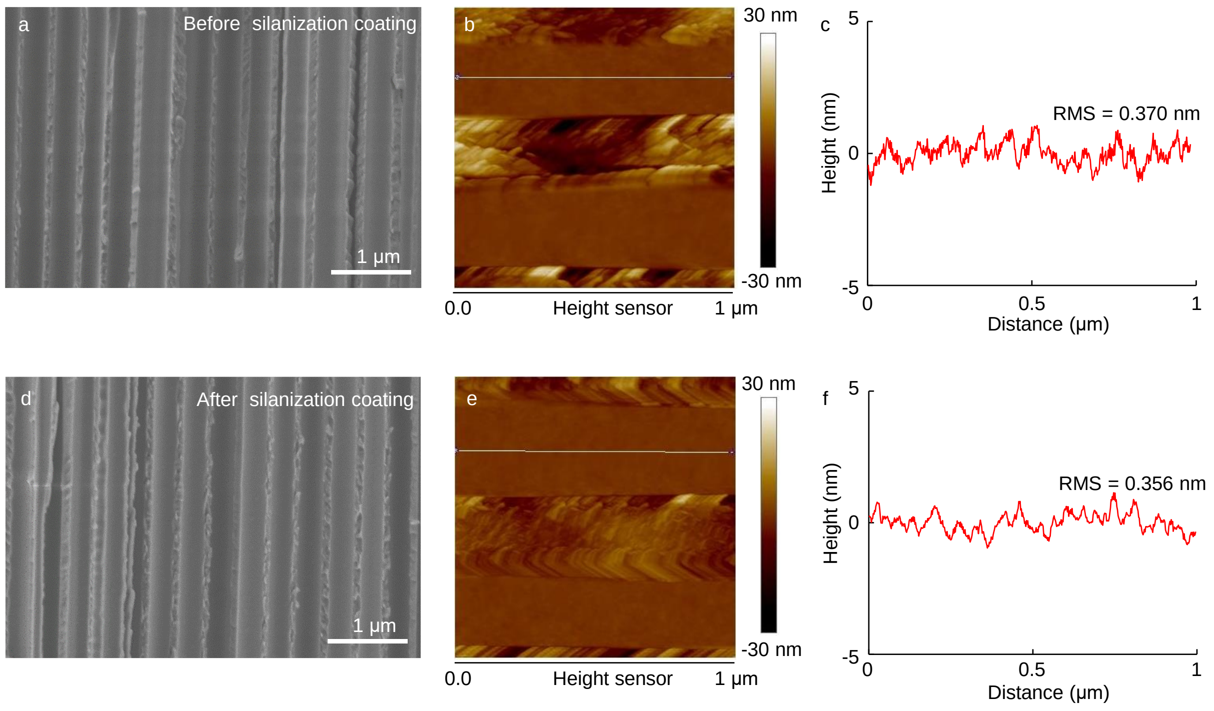

Supplementary Fig. 2 | Characterizing the surface roughness of nanomold walls before (**a-c**) and after (**d-f**) silanization coating by SEM and AFM. The roughness height is described by its root-mean-square (RMS). (**a**, **d**) SEM images of the exposed cavity walls after intentionally break the AAO nanomolds. (**b**, **e**) AFM measured surface topography of the fractured surfaces. (**c**, **f**) Sectional profiles along the white lines as shown in (**b**, **e**). Source data are provided as a Source Data file.

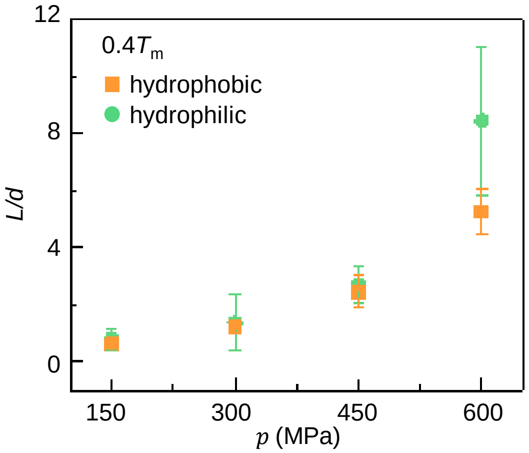

Supplementary Fig. 3 | The length of Bi nanorods versus the molding pressure, where the molding temperature is 0.4T_m (Green dots: before silanization coating, yellow dots: after silanization coating). The error bars are the standard deviation calculated from the length of at least 10 nanorods. Source data are provided as a Source Data file.

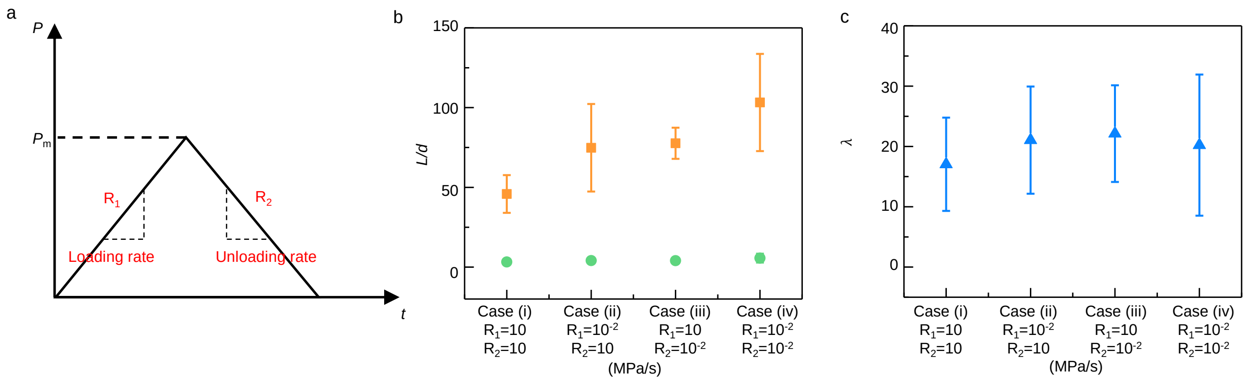

Supplementary Fig. 4 | The effect of loading/unloading rate on the growth of Bi nanorods. The molding temperature and the maximum molding pressure were kept $0.75T_m$ and 300 MPa, respectively. **a**, the loading and unloading procedures. **b**, the green and yellow dots represent the aspect ratio of Bi nanorods molded without and with silanization coated nanomolds, respectively. **c**, calculated λ based on the data in (**b**) and by using eq. (1). The error bars are the standard deviation calculated from the length of at least 10 nanorods. Source data are provided as a Source Data file.

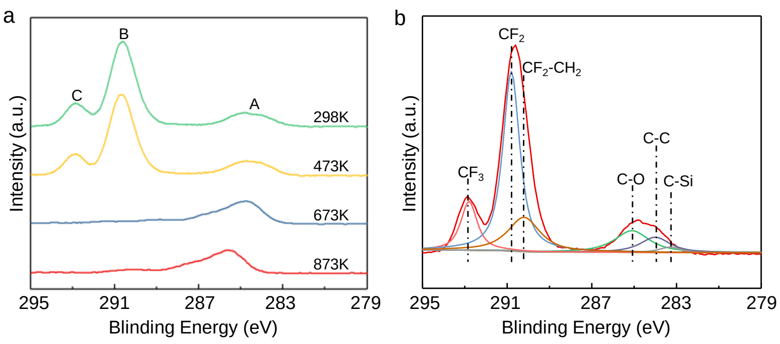

Supplementary Fig. 5 | Thermal stability of silanization coating on AAO nanomolds. All the samples were heated at a target temperature for 10 min. **a,** the XPS results at different temperatures. **b,** high-resolution XPS spectra with constituent fitting. Source data are provided as a Source Data file.

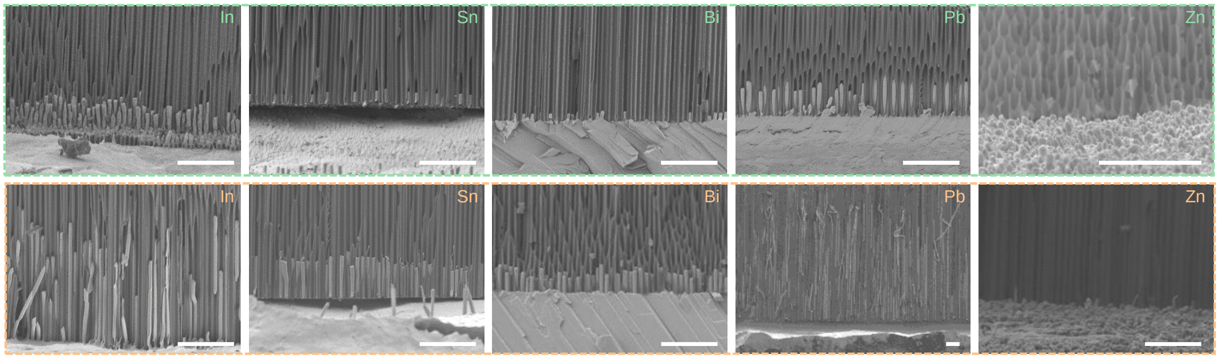

Supplementary Fig. 6 | The influence of crystalline structure on the hydrophobic surface enhanced creep flow. The molding pressure is linearly increased from zero to 300 MPa with a loading rate of 10 MPa/s and the molding temperature is $0.60T_m$. The green and yellow represent molded without and with silanization coated nanomolds, respectively. Scale bars: 5 μm.

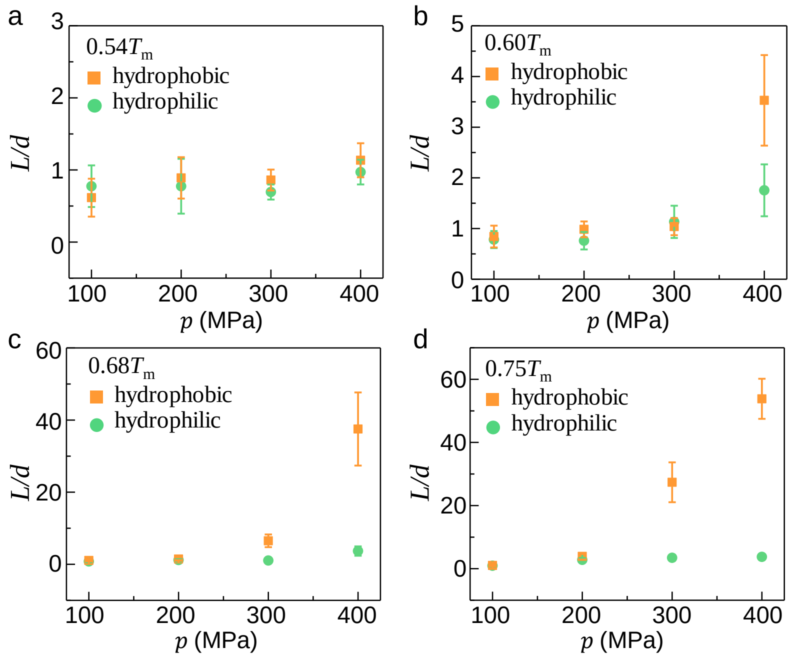

Supplementary Fig. 7 | Temperature dependence of creep flow of Zn enabled by superhydrophobic surface. Measured length of Zn nanowires molded at different pressure and temperature. **a**, 0.54T_m. **b**, 0.60T_m. **c**, 0.68T_m. **d**, 0.75T_m. The error bars are the standard deviation calculated from the length of at least 10 nanorods. Source data are provided as a Source Data file.

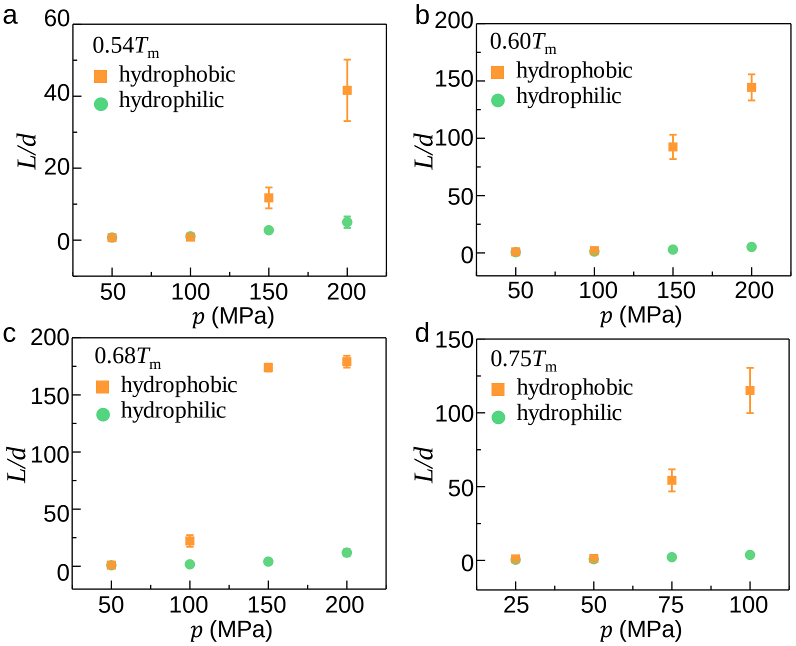

Supplementary Fig. 8 | Temperature dependence of creep flow of Pb enabled by superhydrophobic surface. Measured length of Pb nanowires molded at different pressure and temperature. **a**, 0.54T_m. **b**, 0.60T_m. **c**, 0.68T_m. **d**, 0.75T_m. The error bars are the standard deviation calculated from the length of at least 10 nanorods. Source data are provided as a Source Data file.

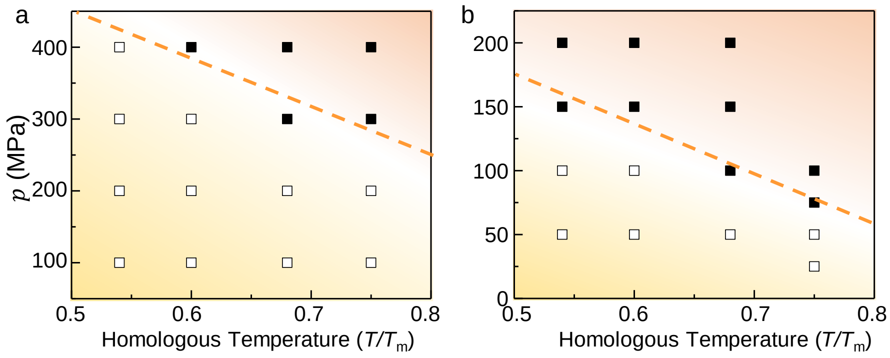

Supplementary Fig. 9 | Temperature dependence of creep flow of Zn and Pb enabled by superhydrophobic surface. **a-b**, the solid and open squares correspond to whether the length difference of Zn (**a**) and Pb (**b**) nanowires molded with pristine and silanization treated AAO nanomolds is significantly distinguishable or indistinguishable, respectively. The dashed line is fitted based on eq. (3).

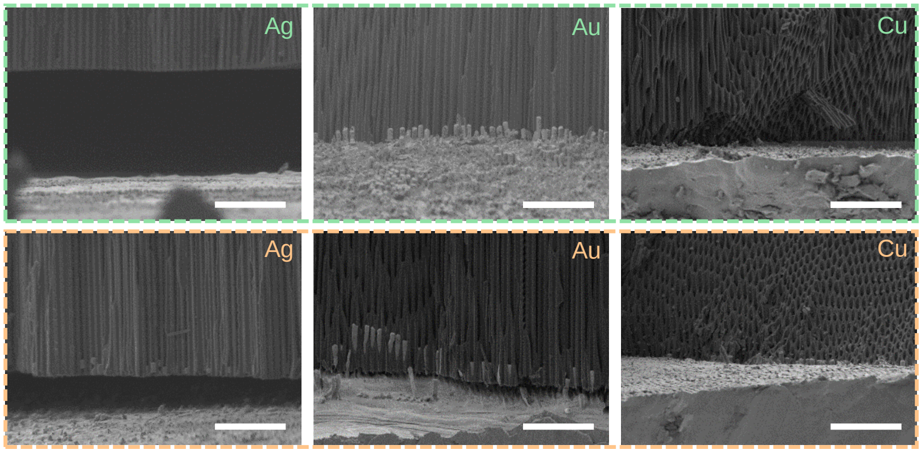

Supplementary Fig. 10 | The length of high melting point metal nanorods molded with pristine and silanization coated nanomolds, respectively. The molding temperature and pressure were $0.6T_m$ and 300MPa, respectively. The green and yellow represent molded without and with silanization coated nanomolds, respectively. Scale bars: 5 μm.

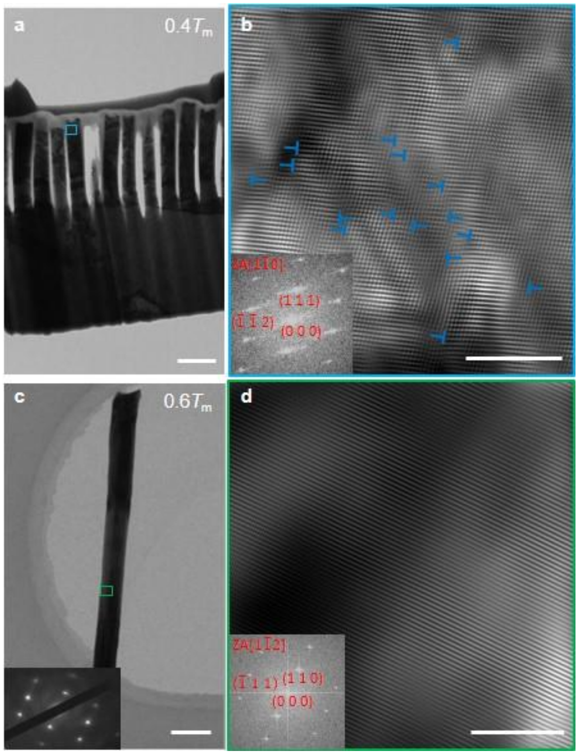

Supplementary Fig. 11 | TEM characterization of Ag nanorods molded at 0.4T_m and 0.6T_m, respectively. a, SEM image of Ag nanorods molded at 0.4T_m and then cut by FIB. Scale bar: 1 μm; b, HR-TEM image of the Ag nanorod at the location denoted by the blue green dot in (a) after inverse FFT of the inset diffraction pattern. Scale bar: 5 nm; c, TEM image of a Ag nanorod molded at 0.6T_m. Scale bar: 200 nm; d, HR-TEM image at the location denoted by the green dot in (c) after inverse FFT of the inset diffraction pattern. Scale bar: 5 nm.

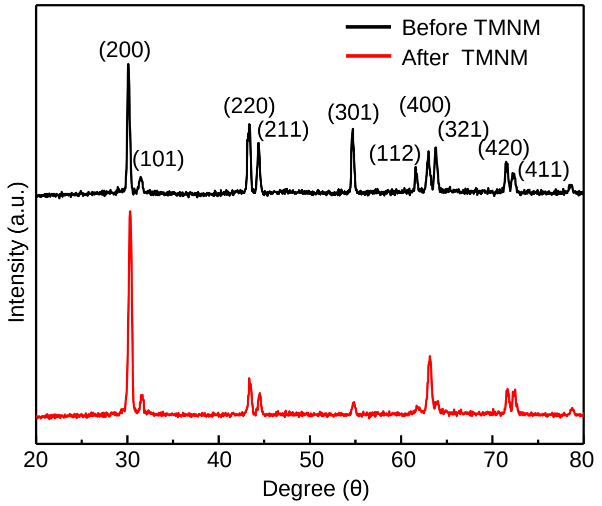

Supplementary Fig. 12 | X-ray diffraction characterization of Sn substrate before and after TMNM, the molding pressure and temperature were 300 MPa and 0.75T_m, respectively. Source data are provided as a Source Data file.

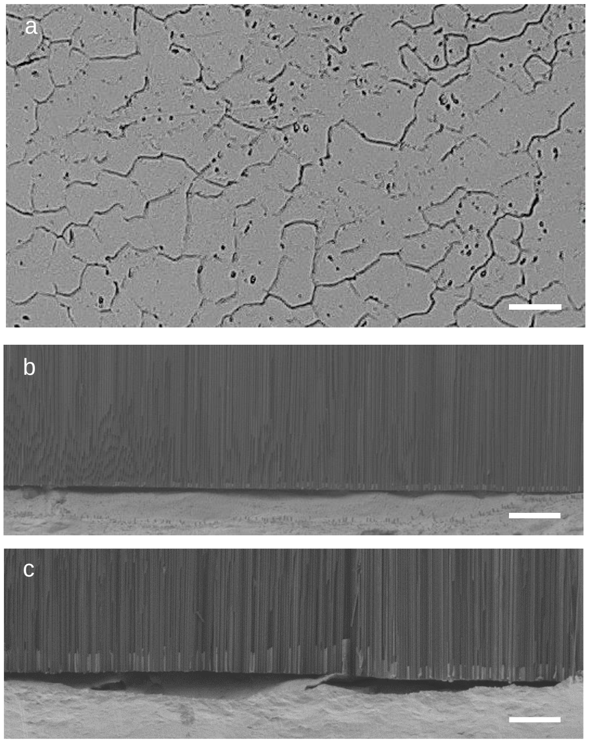

Supplementary Fig. 13 | The effect of polycrystalline substrate. **a,** typical polycrystalline structure of Sn. **b-c,** molded Sn nanorods using pristine **(b)** and silanization coated **(c)** AAO nanomolds. The field should be much larger than the grain size. The molding temperature and pressure are 0.6T_m and 300 MPa, respectively. Scale bars: 10 μm.

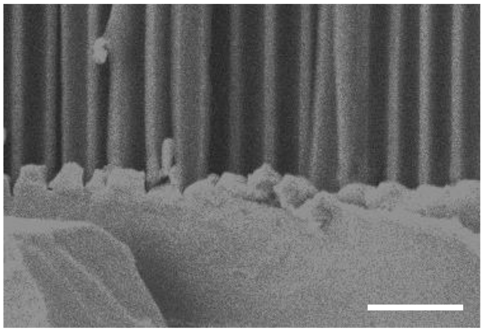

Supplementary Fig. 14 | Determination of the initial length (L_0) of Bi after pre-compression molding. The molding temperature was 200 ℃. The maximum loading force is 1.5 kN. Scale bar: 1 μm.

Table S1 Sample surface relative atomic concentration (%)

Element	298K	473K	673K	873K
C	19.30	18.92	8.55	8.14
O	21.48	22.6	56.31	60.41
F	45.95	44.12	5.23	0.78
Al	11.61	12.56	26.77	27.16
Si	1.66	1.80	3.14	3.51

Table S2 Comparison of the increase of average creep rate of various metals with the change of surface wettability (Supplementary Fig. 6)

Metals	Creep rates (μm/s)
In	0.269
Sn	0.084
Bi	0.054
Pb	1.415
Zn	0.003
